# Effect of probiotic supplementation on organic feed to alternative antibiotic growth promoter on production performance and economics analysis of quail

**DOI:** 10.14202/vetworld.2017.1508-1514

**Published:** 2017-12-25

**Authors:** W. P. Lokapirnasari, A. R. Dewi, A. Fathinah, S. Hidanah, N. Harijani, Soeharsono, B. Karimah, A. D. Andriani

**Affiliations:** 1 Department of Animal Husbandry, Faculty of Veterinary Medicine, Airlangga University, Indonesia; 2 Magister of Veterinary Agribusiness, Faculty of Veterinary Medicine, Airlangga University, Indonesia; 3 Department of Veterinary Public Health, Faculty of Veterinary Medicine, Airlangga University, Indonesia; 4 Department of Veterinary Anatomy, Faculty of Veterinary Medicine, Airlangga University, Indonesia

**Keywords:** antibiotic growth promoter, economic analysis, probiotic, production performance, quail

## Abstract

**Aim:**

The purpose of this study was to know the production performance and economic analysis in quail which use probiotic supplementation to alternate antibiotic growth promoter (AGP) to feed consumption, water consumption, egg production, egg mass, feed conversion, and feed efficiency.

**Materials and Methods:**

About 240 quails (*Coturnix coturnix japonica)* at 14 weeks of age were completely randomized into four treatments, each treatment consisted of six replications and each replication consisted by 10 heads. The treatment was T0 (organic feed without AGP and without probiotic), T1 (organic feed + 0.001% AGP), T2 (organic feed + 0.005% probiotic in feed), and T3 (organic feed + 0.005% probiotic in drinking water). The probiotic consist of 1.2×10^5^ CFU/g of *Lactobacillus casei* and *Lactobacillus rhamnosus*.

**Results:**

The results showed that the probiotic supplementation both in feed and water give a significant impact to feed consumption, water intake, feed conversion, feed efficiency, and quail day production, but no statistical difference of egg mass. The T3 also show the most profitable business analysis, which has the best result in income, profit, break-even point, return cost ratio, benefit-cost ratio, and return on investment.

**Conclusion:**

It can be concluded that giving 0.005% probiotic in drinking water to get the best egg production and profit.

## Introduction

Quail is one of the popular livestock for being able to the needs of the animal protein for the society [1]. More than 40 years, the farmers already use the antibiotics as a feed additive in feed, used as a growth promoter in a small amount but can improve feed efficiency [2]. The overuse of antibiotics may be harmful, which can cause quail resistance to pathogenic microorganisms and can cause residues in meat and eggs that can be very harmful to the consumers [1].

Antibiotics were mostly used in the ration of broiler chicken. The probiotics suggested to alternate the antibiotics [3]. Probiotics are non-pathogenic microorganisms when consumed in certain quantities can provide health benefits [4]. Probiotics are defined as live microbial feed supplement which provides a good effect to the host through increased intestinal microbial balance [5,6].

Probiotics stimulate the growth of beneficial microorganisms and decrease the number of pathogenic microorganisms by increasing the intestinal microbial balance in the host. Probiotic consumption may reduce the risk of gastrointestinal diseases by stimulating beneficial microorganisms [5,6].

Lactic acid bacteria are one of the bacteria that have potential use as probiotics. Lactic acid bacteria can survive by forming colonies of the intestine and can also produce lactic acid and bacteriocins. Lactic acid bacteria consist of several genera of bacteria belonging to the family of Firmicutes [7]. Lactic acid bacterial isolates of the genus *Lactobacillus* are generally potential as probiotic agents which beneficial to human and animal health [8]. *Lactobacillus casei* has both probiotic characteristics and antibacterial activity against different pathogens and can be used as potential functional probiotics in feed [9].

Probiotic treatment may be given through feed and water, then expect that the consumption of probiotics can improve the efficiency of feed and water, then increase egg production and can reduce the cost of production. Through economic analysis with parameters such as the total costs, revenue, profit, break-even point, return cost ratio, benefit-cost ratio, net profit margin, and return on investment, it can be estimated that whether giving quail probiotics in feed and drinking water will be more profitable. The aim of this study was to know the quail farm economics analysis which uses probiotics containing *L. casei* and *Lactobacillus rhamnosus* to alternative antibiotic growth promoters (AGPs) to feed consumption, water consumption, egg mass, egg production, feed conversion, and feed efficiency.

## Materials and Methods

### Ethical approval

There was no need of ethical approval because samples were collected as per standard collection methods without any harm or stress to the animals.

### Study area and farm management

The research was conducted in the Faculty of Veterinary Medicine, University of Airlangga, Surabaya, Indonesia. 240 heads of 14-week-old quail were randomized into four treatments (T0, T1, T2, and T3), each treatment consisted of six replications and each replication consisted by 10 heads, as follows:

T0: Control, 100% organic feed
T1: Organic feed + 0.001% AGP in feed
T2: Organic feed + 0.005% probiotic in feed
T3: Organic feed + 0.005% probiotic in drinking water.

Organic feed is a feed composed of protein feedstuff and energy sources that are formulated according to the needs of the quail. In the organic feed is not added antibiotics but the nutrient value in feed is suitable to fulfill nutrient requirement of quail. A total of 0.001% of AGP were mixed in feed (T1), 0.005% of probiotic (T2) in concentration 1.2×10^5^ CFU/gram, dissolved in 995 mL of water (free chlorine and other antiseptics), and then allowed to stand for 24 h without aeration. A total of 1 L of probiotic solution sprayed evenly into 100 kg of feed and then left the feed dry, so the probiotics absorbed well in the feed, then the feed ready to be given. 0.005% of probiotic (T3) dissolved in 995 mL of water (free chlorine and other disinfectants), then allowed to stand for 24 h without aeration. A total of 1 L of probiotic solution are mixed into 200 L of water, then stir evenly and drinking water ready to be given. The quail was fed twice daily at 7 am and 5 pm. Feed is given *ad-libitum* in mash form. Water was also provided *ad-libitum*.

### Data collection

Collecting data for feed intake are reduced the amount of feed given amount of feed which not consumed. Water consumption is calculated from the amount of water given the reduced with the amount of water which not consumed. Feed and water consumption is calculated every week during the 4 weeks of treatment.

Quail eggs are harvested every day. Eggs are also weighed to quantify the egg mass. Egg production is calculated in quail day production (QDP) that the number of eggs produced per day divided total of females quail in the population and then multiplied by 100%. Feed conversion was calculated from the amount of consumption divided by the weight of the eggs produced and feed efficiency calculated by weight of the eggs produced compared to the amount of consumption. The calculation of the production performance using the following formula:

- QDP
  QDP=Egg production a day/total of female quails×100%
- Feed conversion
  Feed conversion=Feed intake (g)/average of egg mass (g)
- Feed efficiency
  Feed efficiency=Average of egg mass (g)/feed intake (g)

### Statistical analysis

Data analysis was performed using analysis of variance (ANOVA). If the result are significantly different then continued with Duncan’s multiple range test [10]. Statistical analysis using SPSS for Windows 21. 0. Economic analysis and financial data analyze descriptively. Economic analysis calculated with the following formula:

- Production cost
  Total cost=Fixed cost+Variable cost
- Revenue
  Revenue=Price each item×Total of production
- Profit
  Profit=Total of revenue−Total of cost
- Break-even point (BEP)
  BEP production=Total of cost/price of selling
  BEP price=Total of cost/Total of production
- Return cost ratio (R/C ratio)
  R/C=Total of revenue/Total of cost
- Benefit-cost ratio (B/C ratio)
  B/C=Total of profit/Total of cost
- Return on investment (ROI)
  ROI=Net profit/Total of asset×100%
- Net profit margin
  NPM=Net profit/Revenue×100%

## Result

### Effect on feed consumption and water intake

The results of ANOVA 5% can be seen that the addition of probiotics through feed and water, affect to the feed intake and water intake (p<0.05). Furthermore, on feed intake and water intake after Duncan’s multiple range test then can be found that T2 and T3 showed the highest significantly different to controls. The results of feed and water consumption can be seen in Table-1.

**Table-1:** Feed consumption and water consumption of quail in 4 weeks of treatment.

Treatment	Feed consumption (g/quail/day)±SD	Water consumption (ml)±SD
T0	23.0087^b^±0.7519	60.8853^c^±5.3429
T1	23.2009^b^±0.8803	58.7833^b^±2.4270
T2	21.7502^a^±1.7319	56.9523^a^±1.2183
T3	21.6528^a^±0.0181	57.0463^a^±3.2451

SD=Standard deviation,

a, b, c Means having different superscripts within the same columndiffer significantly (p<0.05)

### Effect on feed conversion ratio and feed efficiency

The results of ANOVA 5% can be seen that the addition of probiotics through feed and water affect feed conversion ratio and feed efficiency (p<0.05). Furthermore, after Duncan’s multiple range test it can be seen that the T2 and T3 showed the highest significantly different to controls. The result of the conversion and feed efficiency can be seen in Table-2.

**Table-2:** Feed conversion and feed efficiency of quail in 4 weeks of treatment.

Treatment	Feed conversion±SD	Feed efficiency±SD
T0	2.1139^b^±0.0829	47.5198^a^±1.9975
T1	2.1385^b^±0.0758	46.8907^a^±1.7360
T2	2.0113^a^±0.1477	50.0792^b^±3.7523
T3	1.9984^a^±0.0457	50.0964^b^±1.1482

SD=Standard deviation,

a, b Means having different superscripts within the same column differ significantly (p<0.05)

### Effect on QDP and egg mass

Results of ANOVA 5% can be seen that the addition of probiotics through feed and water affect to QDP (p<0.05), but does not give effect to the egg mass (p>0.05). After Duncan’s multiple range test it can be seen that the T3 showed the highest significantly different to controls. The result of the conversion and feed efficiency can be seen in Table-3.

**Table-3:** Quail day production and egg mass of quail in 4 weeks of treatment.

Treatment	Quail day production (%)±SD	Egg mass (g)±SD
T0	55.7837^a^±3.03104	10.9112^a^±0.34201
T1	55.8847^a^±3.37765	10.8524^a^±0.16868
T2	59.7025^b^±1.22073	10.8085^a^±0.12138
T3	69.6704^c^±2.80250	10.8397^a^±0.21756

SD=Standard deviation,

a, b, c Means having different superscripts within the same column differ significantly (p<0.05)

## Discussion

### Effect on feed consumption and water intake

Results showed that T1 was not significantly different with T0, because the energy and protein content of the feed in the same amount on the treatment, so the feed intake did not differ. The protein content and energy consumption affects to the amount of feed [11]. In addition, many things also affect feed intake, genetic factors such as body weight, strain, age, sex, and the energy content in the diet.

T2 and T3 show the highest significant or give the highest influence. Higher concentration of probiotic given the higher bacteria contained in it, and then the quail will be more efficient in consuming feed. The addition of beneficial microbes for animal, preventing the growth of harmful microbes in the digestive tract so can improve the digestion of feed and can minimize feed consumption [12].

The feeding of viable *Lactobacillus* at 1100 mg/kg (4.4×10^7^ CFU/kg) increased daily feed consumption, egg size, nitrogen, and calcium retentions [13]. However, in this research, supplementation of 0.005% probiotic contains *L. casei* and *L. rhamnosus* that could decrease feed consumption from 23.0087 g/quail/day to 21.6528 g/quail/day, improve feed conversion from 2.1139 to 1.9984 and no significant difference on egg mass. According to Shareef and Al-Dabbagh [14] reported that probiotics (*Saccharomyces cerevisiae*) supplementation of broilers had significantly increased feed consumption. Reviews these results indicated resources to the prominent role of cecal microbiota in the feed efficiency of chickens and suggested plausible uses of *Lactobacillus* to improve the feed efficiency of the host [15]. However, different studies [16] reported the feed consumption was not affected by dietary probiotic supplementation. Results from a study by Babazadeh *et al*. [17] indicated resources probiotics that did not have any significant positive effect on broilers feed consumption and feed conversion.

Water consumption showed that the highest significantly different at T2 and T3, this is due to many factors. Water consumption of animals also depends on other factors, such as activity, environmental temperature, and dryness of the feed, which require much water and relative humidity [18]. Nutritional status, duration of starvation and the relationship between water consumption and animal feed could be used as one of the factors that may affect the responses of animals to be considered [19]. It is important that farms are equipped to provide adequate water volume for optimal development. The fact that water consumption has increased significantly over the past 10 and 20 years is evidence that farm water systems may need to be evaluated to ensure drinking systems have kept up with the changing water needs [20]. This result contrasts [21] that the rise in feed and water consumption is recorded in laying hens fed with probiotics mixed liquid culture containing two types of microorganisms, *Lactobacillus* and *Bacillus* species.

### Effect on feed conversion ratio and feed efficiency

T2 and T3 showed significantly different results of the highest influence on the conversion and efficiency, because the probiotics can enhance microbial activity and digestibility in the quail digestive tract, with the increasing number of population of microbes in the digestive tract, the absorption of feed substances become larger and more effective, which affect to feed efficiency [11].

In line with the research [22] that the use of probiotics can improve feed efficiency during the study. These results are also consistent with studies by Sathya and Muragian [23] that probiotics can improve feed intake and decrease the feed conversion significantly. The results are also consistent with studies by Patel *et al*. [24] the dietary supplementation of probiotics at 100 g/ton of feed significantly enhanced body weight gain along with better feed conversion ratio and profit. The *Lactobacillus* sp. strain was able to colonize the intestinal tract and feed and remain at a high concentration of 10^7^ and 10^6^ CFU/g, respectively. It produced several enzymes, which might have contributed to the greater weight gain and lower feed conversion in the supplemented animals [25].

### Effect on QDP and egg mass

T2 and T3 showed significantly different results and T3 showed the highest significantly different in QDP. Through the work of probiotics in the small intestine and colon, by pressing the pathogenic bacteria and stimulate the growth of good bacteria that will increase the capacity of absorption and digestibility of protein, so can increase egg production [23]. Protein is one of the basic ingredients in the feed. Protein is needed for body growth of poultry, changing the damaged tissue, and also for production [26]. Protein is an egg-forming element [27]. High protein content in the feed can increase egg production [28]. Egg production, however, widely varied attributed which could be partly to genetic and non-genetic factors [29].

In line with the past study [11], the addition of *Lactobacillus salivarius* as much as 7 g per kilogram of feed provides a very real effect on the production of eggs in quails. These results also consistent with past study [30] that *L. fermentum* affects egg production. *Lactobacillus* can be used as probiotics in livestock that works to increase the productivity of livestock [31].

Probiotics do not give significant effect to the egg mass. The factors that cause variations in egg mass are the natural pattern of egg production, feed, management, and also other factors related to genetics. Egg mass is genetically inherited. Environmental influences such as pen environment, parent body size, the maturity stage, age, medications, type of feed, the amount of feed, and food substances in the diet such as the adequacy of protein and linoleic acid greatly affect the of the egg mass [29]. Egg production in a relationship with genetic selection makes today’s egg production quails quite different from reviews those of a decade ago [30].

A similar result was observed by Kulsum *et al*. [31] that *L. fermentum* does not give effect to the egg mass. Another study [32] showed that probiotic did not affect egg mass in Japanese quails. Another study also indicates that the use of probiotic does not necessarily indicate a positive response. The use of *Bacillus subtilis* (CH201) and *Bacillus licheniformis* (CH200) at multiple concentrations, respectively, 0, 400, 1000, and 2000 g/ton of feed containing 0, 1.28×10^6^, 3.2×10^6^, and 4.6×10^6^ CFU/g of feed concentration no significance difference in feed consumption, egg production, and egg weight (p>0.05) [33].

The results of the study [34] indicate that supplementation with commercial probiotics and prebiotic does not show significant results to the increase in percentage of fertile egg and hatchability. Another study [35] indicates that the probiotics supplementation at 500 g/ton on commercial feed can improve feed intake and feed conversion, while on egg production and egg mass there is no significant difference.

A review of past studies has revealed that the effective administration dosages of probiotics vary greatly and is dependent on the strains used and the clinical characteristics of subjects, such as lipid profiles. Although probiotics have been delivered in the range of 10^7^ to 10^9^ CFU/day in animals [36].

### Financial analysis

#### Investment cost

The investment costs are those costs incurred in the 1^st^ year of the project consisting of pen, equipment, and land lease. Pens are used for the production of quail which ready to lay eggs. Land lease fees consist of space lease, electricity, and water for a month. Cost of equipment consists of the feeding, drinking, wire, sprayer, etc. The investment cost of each treatment is equal because the cost of the pen and land lease fee of each treatment for all inputs which used in this analysis is real. Input price is the price which prevailing at the time of the study. The investment costs can be seen in Table-4.

**Table-4:** Fix cost of treatment.

Description	Economics age	T0	T1	T2	T3
Pen depreciation (IDR)	18 months	13,889	13,889	13,889	13,889
Tools depreciation (IDR)	18 months	1,667	1,667	1,667	1,667
Transportation (IDR)	-	15,000	15,000	15,000	15,000
Total (IDR)		30,556	30,556	30,556	30,556

IDR=Indonesian rupiah

#### Fixed cost

Fixed costs are costs that are not influenced by the size of total product, and it is equal every year. The fixed costs consist of pen and equipment depreciation. Pen and equipment depreciation costs are calculated with depreciation formula that divided the economic life of the investment costs, which the equipment has an economic life of 18 months. There are no electricity costs because it is already included in the land lease fee and there are no labor costs because everything is done by the researcher. Fixed costs have the same amount of treatments because the equipment used is the same. Fixed costs can be seen in Table-5.

**Table-5:** Variable cost of treatment.

Description	T0	T1	T2	T3
Vitamin (IDR)	2,000	2,000	2,000	2,000
Feed (IDR)	126,272	127,326	119,365	118,830
Feed additive (IDR)	0	5,000	400	800
Quail (IDR)	17,778	17,000	17,000	17,000
Total	146,050	152,104	139,543	139,408

IDR=Indonesian rupiah

### Variable cost

Project time is based on a long research, which is 4 weeks so that the variable costs are calculated on the cost of production for 4 weeks. Cost of production consists of fixed costs and variable costs [37]. Variable costs are costs which amount is influenced by the amount of product. Variable costs consist of the cost of transport, vitamins, feed, feed additives, and quail. The difference showed in variable cost, on the feed and feed additive costs.

In poultry production, total expenses greatly influenced by the price of feed that can reach up to 70% of the total cost [38]. High cost of production especially the cost of feed ingredients as their major constraint [39]. The cost of feed is calculated from the total of feed intake of each treatment for a month multiplied by the price of feed is Indonesian rupiah (IDR) 4,900 per kilogram feed. The precise information on feed conversion is important to calculate each bird feed consumption [40]. The results showed that the lowest feed costs at T3 IDR 118,830.

Cost of feed additives is the amount of feed additive given for 1 month multiplied by the price of feed additive, which is IDR 40,000 per 500 g of AGPs (T1) and IDR 25,000 per 100 grams of probiotic (T2 and T3). T0 is not given a feed additive so that the cost of feed additive is IDR 0. The highest cost of feed additive on TI which uses AGPs IDR 5,000 and the lowest feed additive cost in the T2 (0.005% probiotics in feed), IDR 400. Vitamin given once a week for a total administration for 4 weeks was 10 g per treatment. The cost of vitamins and quail each treatment has the same amount as the number of birds that use the same.

The results showed that the lowest variable cost or the most efficient at T3 is IDR 139,408, while the highest variable costs in T1 IDR 152,104 due to the high cost of feed additives. Variable cost of research can be seen in Table-5.

### Revenues and profits

The flow of revenues is from the sale of quail eggs and sale of faces. The total of quail is 40 per treatment and then calculated with the total number of eggs produced during 4 weeks. Quail eggs sold at IDR 270 per item. While the feces sold at IDR 5,000 per sack, each sack contained of 50 kg.

The results showed that the highest total income in the T3 (addition 0.005% of probiotics through drinking water) IDR 240,980 and affect the profits, where T3 get the highest benefit IDR 71,016, this is due to higher production of eggs in T3. Profit condition happens if the income is greater than the cost of production [41]. Total revenues and profits can be seen in Table-6.

**Table-6:** Total of revenue and profit each treatment.

Description	T0	T1	T2	T3
Egg production (egg)	730	721	700	874
Egg’s sell (IDR)	197,100	194,670	189,000	235,980
Feces (IDR)	5,000	5,000	5,000	5,000
Total of revenue (IDR)	202,100	199,670	194,000	240,980
Total of cost (IDR)	176,605	182,659	170,098	169,963
Profit (IDR)	25,494	17,010	23,901	71,016

IDR=Indonesian rupiah

### Economic analysis

From the results indicate that best BEP production and BEP price is T3 is only with the production of 629 eggs at a price of IDR 194/item does not give a profit or a loss. BEP value can indicate the level of production and the price of what a business does not provide a profit nor a loss [41].

Based on the results, the best R/C ratio shown in T3 treatment (1.417). The criteria to the calculation of business efficiency, namely, when the R/C ratio <1, then the business is said to be inefficient, when the R/C ratio is equal to one then the business is said to be unprofitable or no damage and if the R/C ratio is more than one then said to be efficient or beneficial [41]. Hence, the business is worth it because it has a value of more than 1. Net R/C 1.417 means that every IDR 1, - the costs over the life of the project resulted in IDR 1,417 revenue.

Based on the results of B/C ratio, the best shown in T3 0.417. The criteria against business efficiency calculation that if B/C ratio is <0.1 then the business is said to be inefficient or harmful, when the B/C ratio equal to 0.1 then it is not profitable or do not harm and if the B/C ratio more 0.1 the business to be efficient or beneficial [41]. Hence, the business is beneficial because it has a value of more than 0.1. Net B/C is equal to 0.417 means that every IDR 1, - the costs over the life of the project resulted in IDR 0.417 profits.

While that the best return on investment value on T3 which is 15.887%, so we can say the business has the highest number of assets that can be used. Business analysis is shown in Table-7.

**Table-7:** Economics analysis each treatment.

Description	T0	T1	T2	T3
Break-even point (product)	654	676	629	629
Break-even point (price)	241	253	242	194
r/c ratio	1.1445	1.0931	1.1405	1.4178
B/C ratio	0.1445	0.0931	0.1405	0.4178
Return on investment (%)	5.7035	3.8055	5.3471	15.8874

## Conclusion

Probiotics give a significant effect on feed intake, water intake, feed efficiency, feed conversion, and QDP, but give no significant effect on egg mass. Best production performance results and most profitable economic analysis are the addition of 0.005% probiotic through drinking water. So that farmers can give 0.005% of probiotics through the water to get the best production performance and profit.

## Authors’ Contributions

WPL designed the research, conducted the experimental work. WPL, SH, S, and NH analyzed and interpreted the data and drafted the manuscript. ARD, AF, BK, and ADA participated in the collecting data, analysis, and interpretation of data and drafting of the manuscript. All authors read and approved the final manuscript.
